# Use of a fictitious community-based virtual teaching platform to aid in the teaching of pharmacy practice skills: Student perspectives after initial implementation

**DOI:** 10.1186/s40545-016-0077-3

**Published:** 2016-09-22

**Authors:** Louise E. Curley, Maureen McDonald, Trudi Aspden

**Affiliations:** 1 School of Pharmacy, Faculty of Medical and Health Sciences, The University of Auckland, Private Bag 92019, Auckland, 1142 New Zealand; 2 School of Pharmacy, Faculty of Medical and Health Sciences, University of Auckland, NZ Private Bag 92019, Auckland, 1142 New Zealand

**Keywords:** Simulation, Community pharmacy, Virtual community, Teaching platform, Vignettes

## Abstract

**Background:**

Providing patient-centred care requires pharmacy students to learn *how to* interact effectively and understand individual differences that can influence patients’ health. The School of Pharmacy at The University of Auckland, New Zealand (NZ), developed a virtual teaching platform, called NZ Pharmville, which consisted of twenty-one community-based patients who are members of six families; each family had a video vignette associated with it. Bachelor of Pharmacy (BPharm) students, enrolled in the third year pharmacy practice course, were able to view these recorded vignettes as part of their weekly pre-laboratory work for the course. All the clinical cases within the course were based on this community, with the aim of increasing the realism in the practical sessions and increasing patient-centred learning. This study aimed to explore the perspectives of pharmacy students regarding the integration of this virtual community into a third year undergraduate pharmacy practice course.

**Methods:**

An anonymous, voluntary survey which consisted of twenty-one items, 13 requiring a Likert scale response and 8 requiring free text responses, was distributed to all students who had completed the third year pharmacy practice course. The responses to the questions were collated and analysed. Responses to questions one to thirteen were recorded in Excel, and results were presented as the combination of strongly agree and agree, strongly disagree and disagree and neutral. Responses to free text questions were read multiple times before being coded by two members of the research team into broad themes aligned to the overall aims of the evaluation.

**Results:**

Eighty-six (80.4 %) of the eligible students completed the survey and the majority of responses were positive towards the benefits of using the virtual community in the course. Responses indicated that many of the students found the integration of the virtual community to be useful preparation for their practical sessions and the majority of students felt that the vignettes made it easier to develop empathy for the patients rather than reading about them.

**Conclusion:**

The use of virtual communities, for example, NZ Pharmville, show promise as a platform to aid in teaching and learning. The resources in NZ Pharmville allow students ongoing access to patient video clips that attempt to depict a real life situation, and enable students to engage with the fictional characters. The virtual community provided an educational experience which was well received by students. This teaching method appeared to promote active patient-centered learning and allowed students to reflect on and revisit these skills on a weekly basis.

## Background

An essential skill, of a practising pharmacist, is effective communication. Pharmacists must build rapport with patients and interact with diverse groups of individuals, including patients, their family members and other health care professionals. Patient-centred care has been defined by the Institute of Medicine as: “care that is respectful of and responsive to individual patient preferences, needs, and values and ensuring that patient values guide all clinical decisions” [1]. Providing patient-centred care therefore requires pharmacy students to learn how to interact effectively, as well as understand individual differences that can influence patients’ health [2].

Many pharmacy education councils are encouraging teaching in a manner that centers around the patient, for example, The Accreditation Standards and Guidelines set by the Accreditation Council for Pharmacy Education (ACPE) [3] and the educational outcomes of the Center for the Advancement of Pharmacy Education (CAPE) [4] both promote the application of knowledge in a patient-centred manner. In addition, Standard 12 of the ACPE Accreditation Standards promotes the use of patient simulation through the use of educational technologies [5].

Lectures have traditionally been the principal teaching method used at universities, and students often view lectures and the recall of knowledge as the core of their education. The nature of lectures means that they do not easily allow for producing the depth of learning needed to develop core skills [6]. Lecture theatres are often constrained spaces, which limits the range of learning activities that can occur during a lecture, in particular the development of skills [6]. Teaching the application of concepts can therefore be challenging in pharmacy education. Active learning theory acknowledges that hands-on training experiences are necessary to move students from shallow to deeper levels of learning by allowing them the opportunity to practice and receive feedback on their application of basic skills and knowledge [7]. The professional practice of pharmacy is based on skills, attitudes and knowledge; consequently, active learning is considered an essential component of pharmacy curricula [7]. Kolb’s learning cycle is a four stage active learning process describing how knowledge is acquired and embedded through reflective observation, abstract conceptualisation, active experimentation and concrete experience. Kolb argues that an individual must progress through all four of these stages for new knowledge to become embedded [8].

Typically, students enrolled in pharmacy education courses are presented with opportunities to practice core skills, including clinical, dispensing, and counselling skills, in the classroom by working through patient scenarios. In order to routinely provide patient-centred care students need to be able to interact effectively with a diverse range of patients. This requires that students appreciate that each patient is unique and includes consideration of many factors including knowledge and familiarity with medicines and medical conditions, health beliefs and family support, which can all influence how individuals view and manage their health. Whilst ideally these scenarios would be taught in small groups and involve the use of “real” patients or patient-actors, budget and logistical constraints often mean this does not regularly occur within curricula [9].

In an attempt to create cost effective and realistic learning environments, a range of different technologies have been utilised. Simulation based learning has been used in healthcare education since the early 1970s [10]. Simulation as a teaching tool includes simulated patients, standardised patients, human simulators, computer based learning simulations and virtual reality patients [10]. Simulation-based learning allows students to practice their skills and apply their knowledge in a realistic, safe and controlled environment [11, 12]. Simulation has been shown to be an effective learning tool and there have been many favorable reports where comparisons have been made with more traditional teaching methods [7, 13–15].

A number of institutions across Europe and the United States of America have implemented various types of virtual patients and evaluated both the educational aspects and student perspectives. Virtual patients have been defined as computer programs that simulate lifelike clinical scenarios in which the learner becomes the health-care professional making therapeutic decisions. Studies have reported that the use of virtual patient based learning and assessment tools result in benefits to both the clinical reasoning and communication skills of students [16, 17]. In addition, learning using virtual patients has been shown to increase the knowledge of health professionals [7, 11, 15].

With the aim of creating more depth and context for case-based teaching using virtual patients, Monash University created a virtual community (Pharmville) which reflected Australian demographics and consisted of several different family groups with different living situations [18]. Research surrounding this initiative was reported by Marriott et al. (2012), which found that using a virtual community as a teaching platform showed a positive response from students and that students were able to “identify with various characters within the community” [18].

At the University of Auckland, pharmacy practice is taught in each year of the Bachelor of Pharmacy curriculum. During the third year of the degree, the pharmacy practice course aims to ensure students can communicate effectively, and safely and professionally dispense a range of prescriptions in a *patient-focused*, ethical and legal manner. The pharmacy practice course is taught to students using online and face to face interactive lectures, Responding to Symptoms (RTS) workshops and dispensing laboratory sessions. The latter two use a mock pharmacy environment which enables students to practice their skills in a safe (for both the student and the public) yet realistic learning environment. There are many benefits of the established methods used in teaching of the dispensing laboratory sessions and RTS workshops, including interactions with tutors, many of whom are practising pharmacists, and the ability to gain experience using the dispensing software programs commonly found in community pharmacies. However, there are also limitations including providing a realistic environment and developing empathy for the fictional characters, as the tutors have to act the role of both the patient and the prescriber.

In order to enhance the realistic environment and provide context to our teaching, we followed the lead of Monash University and developed a community-based virtual teaching platform, consisting of six families and twenty-one individuals accessed by students via the University of Auckland’s learning management system. We believed by creating our own version of this virtual community, we would be able to create a flexible resource for academics to use to aid student learning that reflected a selection of health issues in New Zealand. Benefits to students were anticipated to include the opportunity for repeat access to this resource without restrictions on when or the number of repeat visits and thus the teaching exercises could be continually revisited and repeated. To distinguish this community from that developed by Monash, the community was created to reflect the demographics of a typical suburb in New Zealand and was named NZ Pharmville. All the characters that were created in the New Zealand Pharmville were different to those in the Monash platform.

Through this virtual community we aimed to use technology to promote active and patient-centred learning a) To provide more in-depth context to the patient scenarios that the students are presented with. b) To facilitate the students becoming more aware of other factors that may affect a particular patient’s condition, for example, diet and exercise, to better inform them of their approach and ability to tailor suitable options to manage specific patients. c) To facilitate the students becoming more aware of the epidemiology of the medical conditions studied within the third year of the Bachelor of Pharmacy curriculum.

This study aimed to evaluate students’ perceptions of this teaching intervention. Specifically we wanted to assess whether students perceived NZ Pharmville to be a realistic learning environment and whether students believed that NZ Pharmville was helpful for developing their interpersonal skills when questioning and counselling patients, and aided their preparation for practical sessions. Student views around any improvements which could be made were also sought.

## Methods

### Course Information: the teaching platform

Traditional pedagogy of pharmacy education has evolved, from the traditional face-to-face didactic lectures to include small group practical session teaching and self-directed learning using online resources. Through this teaching platform we incorporated both traditional and new pedagogy to the third year pharmacy practice course.

A series of video vignettes, with one vignette per family in the virtual community, were developed. The virtual community was designed to make student practical sessions more realistic, to allow the discussion of lifestyle concepts that could affect clinical decision-making and counselling, and ensure each laboratory session used a patient-centered approach. Each vignette consisted of a brief introduction to the family in their own home environment. Most family members were present in the vignettes, which also contained descriptions of their family and their lifestyle in a reflective narrative style.

The vignettes were between 2 and 3.5 min in length, and were embedded within the course’s online website. They provide students with the opportunity to visualize and become familiar with the patients and their families in their own time and pace prior to their practical dispensing sessions. The course used the NZ Pharmville virtual community in addition to standard lectures, with the aim of creating a more realistic setting for the practical dispensing and responding to symptoms component of the course. In an introductory lecture, the students were familiarized with how to access to the online video vignettes, and how it was envisaged that the vignettes would be used by students. On a weekly basis, in advance of the next laboratory session, students were informed of the general medical conditions they would be encountering, the medications that would be prescribed and the patient who would be presenting to the pharmacy with a prescription. This gave students the opportunity to review the online family vignettes for any information that was particularly relevant to the presenting patient or their lifestyle. When the students arrived at each practical session they were given the prescription(s) for the presenting patient(s).

### Course Information: learning objectives

There are two main changes with the integration of the virtual community. Firstly, ensuring that all the cases used in the dispensing classes reflect characters in the NZ Pharmville community, and secondly that paper-based cases are now visually represented via video vignettes.

The learning objectives of the practical sessions for the pharmacy students were to communicate effectively and professionally and safely dispense a range of prescriptions in a *patient-focused*, ethical and legal manner. Within each dispensing laboratory session, students evaluated the prescriptions for legal, clinical and cost appropriateness. Consequently the student had to talk to the ‘patient’ and then talk to the ‘prescriber’ to resolve any issues. The students then prepared labels, dispensed the prescription and documented counselling points for the patient. Practicing and academic pharmacists and pharmacy technicians facilitated the sessions and role played being patients and prescribers.

The course allowed students to learn the core knowledge and skills in a standard format, through lectures and readings. The small group workshops and laboratory sessions, with the additional integration of concepts being taught using the virtual community, then allowed the knowledge and skills to be used at the application and evaluation levels of Bloom’s taxonomy [19]. The topics covered included infections, dermatology, gastrointestinal, respiratory, cardiovascular, women’s health, pain, and eye and ear conditions. Students were aware that the skills and knowledge taught in these sessions would be integrated into their end of course objective structured clinical examination (OSCE). The practical dispensing sessions progressed in complexity throughout the semester. One example of a case of moderate complexity was in the respiratory teaching week which is described in Table 1.

**Table 1:** An example of a case study from the NZ Pharmville community

*Case example:*
The patient presented with inadequately managed asthma and a prescription for an asthma reliever inhaler. The patient was a child who was seven years old.
The online video vignette for the family details the family life, where the parents of the child discuss their lifestyle and health beliefs in a narrative manner.
By watching the vignettes, obtaining a medical history and asking appropriate questions the students must ascertain that the reason that the child’s asthma is inadequately managed is due to non-compliance, driven by beliefs that the parents would like to use a more natural alternative to the preventer inhaler.
*Optimal outcome for the teaching session:*
The practical dispensing session requires the students to recognise the problem, discuss with the prescriber, dispense the medicine and provide appropriate counselling to address the issues in a patient centred manner.

### Study design

This study was a cross-sectional evaluation of student perspectives of a virtual community-based teaching platform in a third year pharmacy practice course within the BPharm degree at The University of Auckland. Specifically we investigated the acceptability of the virtual community, student engagement with the families and their use of the vignettes within the course. Ethics approval was granted by The University of Auckland Human Participants Ethics Committee (015932).

Student perceptions were assessed using a questionnaire, developed by two of the researchers. The final questionnaire comprised twenty-one items, thirteen used a five point Likert scale (strongly disagree, disagree, neutral, agree, strongly agree) to determine student opinions while the remaining items required free-text responses (refer to Tables 2 and 3 for question details). The questionnaire was developed to address the questions that the researchers were interested in exploring. Please note that all references to Pharmville in the questions and answers refer to NZ Pharmville.

**Table 2:** Student responses to items 1-13

Statements	Percentage responses (%)		
	Strongly agree or agree	Neither agree or disagree	Strongly disagree or disagree
1. Watching the Pharmville clips prior to laboratory sessions encouraged me to participate more during the dispensing laboratory sessions.	35	37	28
2. The Pharmville video clips make the learning interesting.	45	37	17
3. The Pharmville video clips encourage me to complete the laboratory and workshop pre-work.	45	28	27
4. Pharmville has helped to develop my competence in counselling to patients	44	31	24
5. Pharmville has helped to develop my confidence in counselling to patients.	29	40	31
6. Having the lecture, lab and workshop cases based on the Pharmville families is useful preparation for the labs and workshops.	54	27	19
7. Pharmville has helped me to tailor my patient interview questions and counselling to specific patients and their situations.	56	25	19
8. Pharmville has helped me to focus my decision making and advice more around the patient as opposed to the medicine.	56	32	12
9. I am well prepared for my future role involving interacting with patients.	64	25	11
10. Watching the Pharmville video clips prior to laboratory and workshop sessions is useful preparation for the labs and workshops.	52	29	19
11. Watching the Pharmville video clips makes it easier to develop empathy with the fictional patients than reading a patient description.	65	19	15
12. Watching the Pharmville video clips helped to improve my problem solving skills.	35	42	24
13. Pharmville helped me understand how the content of the BPharm curriculum is relevant to a career in pharmacy.	54	32	14

**Table 3:** Description of questions 14 - 21

Question	Response rate (percentage; *n* = 86)
14. How did you usually use the video clips in PHARMACY 301?	79
15. How did watching the Pharmville clips help you in PHARMACY 301?	64
16. Which families are the most realistic and why?	35
17. Which are the most unrealistic and why?	28
18. Do you have any suggestions for improving Pharmville?	48
19. Do you have any suggestions for where else the Pharmville families could be included in the BPharm programme?	19
20. How do feel that Pharmville and the related clips helped your learning?	35
21. Space for any other comments about Pharmville that you would like to make.	17

### Data collection

Students were informed of the study by an electronic announcement and provided with the participant information sheet 1 week prior to completion via the University of Auckland’s learning management system. Students were then invited to complete the paper-based questionnaire at the end of the final lecture of the course which took place in October 2015. Participation was voluntary and anonymous. It was made clear that participation or non-participation would not affect the students’ course grade. No incentives were given for the students to complete the questionnaire.

### Data analysis

Responses to statements 1-13 were recorded in Excel, and results are presented for the combination of strongly agree and agree, strongly disagree and disagree and neutral. Responses to free text questions were read multiple times before being coded by two members of the research team into broad themes aligned to the overall aims of the evaluation.

## Results

Eighty-six out of 107 students (80.4 %) enrolled in the third year pharmacy practice course of the Bachelor of Pharmacy, at The University of Auckland, completed the survey. Seven of the thirteen statements (questions 7-13) were only answered by 84 students, and one question (question 6), was answered by 85 students. All results therefore are presented as percentages for questions 1-13.

### Likert scale statements

A majority of responding students strongly agreed or agreed with statements 11 (65 %), 9 (65 %), 7 and 8 (both 56 %), 6 and 13 (54 %) and 10 (52 %). Six of the 13 Likert scale items resulted in students responding less than 50 % that they agreed/strongly agreed with the statements; with positive responses ranging from 29 % (item 5) to 42 % (item 12). There were a considerable number of students who responded with a neutral response, i.e. neither agree nor disagree. Negative responses (strongly dis agree or disagree) ranged from 11 % (item 9) to 31 % (item 5).

Refer to Table 2 for a comprehensive description of the Likert scale items and student responses to them.

### Free text analysis

Whilst not all the students provided responses to these questions, most of the responses received provided additional information which was consistent with the quantitative item responses and provided more detail and further insight into student perceptions of the intervention. The questions asked and response rates to each are detailed in Table 3.

#### Use and perceived benefits of viewing the video clips in the course

The responses indicate that most students used the video vignettes to prepare for their workshop and laboratory sessions. Responses related to the course itself indicated that the perceived benefits of the clips to most students enrolled in the course were that they gave context and background information about “patients” that they would be encountering in the dispensing laboratories.

Responding students indicated that they predominantly used the video clips to obtain information about the patient to either inform their patient questioning at the beginning of the practical session or their counselling points at the end of the session. Some students used them for both.

*“I just watch it before lab, so that I have an idea about the patient’s background and what to ask the patient” [respondent 74]*

*“to understand more about the patient-specific problems” [respondent 73]*

*“pre-lab to try and understand the patient’s lifestyle. Prepare individual counselling” [respondent 4]*

Some students reported that the clips assisted with relating to the patient.

*“Help understand that every patient is different and requires different counselling and medication” [respondent 4]*

*“it allowed me to see what patient families were like” [respondent 13]*

*“they help us get to know the patients better. Know about their lifestyle” [respondent 36]*

*“allowed me to connect to the patient so the prescription felt like it was for someone” [respondent 16]*

*“good to be able to relate to a "real life" type situation” [respondent 80]*

*“I knew more about the patient. This better stimulated a real-life situation in terms of dispensing an Rx” [respondent 65]*

Other responses suggest the clips facilitated the development of empathy with the patient for some students.

*“empathy for patients. How their condition effects their lives” [respondent 71]*

*“it gives an insight into a patient life. So it helped with counselling” [respondent 49]*

Some students noted that the clips provided more knowledge about the patient prior to attending the laboratory which enabled them to be better prepared.

*“they prepared me for what was going to happen in the labs” [respondent 56]*

*“it helped me be prepared beforehand” [respondent 77]*

The responses indicated that whilst some students appeared to watch the vignettes throughout the course not all students did. A minority responded that they didn’t watch them at all and some that they only viewed them for the first few weeks of the course.

*“rarely used but when I did it encouraged me to participate more” [respondent 19]*

*“not very much, I would ask the patient the right questions to get the same information” [respondent 43]*

Some students provided reasons given for stopping watching the vignettes which included a perceived lack of integration between the clips and laboratory sessions, a heavy course workload and that they could complete the laboratory classes without watching them.

*“at the start of semester watched the clips and tried to come up with problems they may have. Towards the end of semester I stopped watching them as I felt as though they weren’t integrated into the lab” [respondent 8]*

#### Perceptions of and engagement with the NZ Pharmville community

Students were asked in question 16 and 17 which families they thought were the most and least realistic. This question aimed to see whether a realistic virtual environment was created. There were mixed views amongst the responding students regarding which families and individuals were the most or least realistic. In addition a few positive and negative comments were made about character and family realism in general.

*“I would say they are realistic as each other” [respondent 5]*

*“cant remember names but - the family with the gout patient and the vegetarian wife the man and the grandmother did very well… the pacific family with the man who likes fishing - =)” [respondent 7]*

#### Suggestions for improvements

Suggestions made by students for improving NZ Pharmville included incorporating the clips into the actual dispensing laboratory sessions rather than viewing them as part of their preparation, producing more video clips of existing individuals with more details tailored to specific laboratory classes and for more families to be created.

*“maybe more families” [respondent 57]*

*“tutors should go over the clips with us during a lecture” [respondent 68]*

*“maybe instead of looking at the videos online, integrate these families into the our actual workshop or labs. For me, it felt like it would be better if they were actual patients” [respondent 35]*

## Discussion

The use of virtual patients has been well studied in pharmacy education and they have been shown to be an effective teaching tool which can provide positive student learning experiences [11, 15, 17, 20], however the use and evaluation of virtual communities has been less well documented in pharmacy education.

This study aimed to evaluate student perceptions of the virtual teaching platform, NZ Pharmville. Students reported that the video clips in NZ Pharmville assisted with preparation for practical sessions. Furthermore, students reported that the video clips helped focus their decision-making and advice for the patient. The results also indicated that by having a community of virtual patients there was added realism, shown through students reporting increased empathy (Item 11), and helping them to interact with patients (Item 9). In addition, with respect to adopting a patient-centred approach to learning; the majority of responding students felt that the use of the virtual community also aided them in tailoring both their patient questioning and counselling (Item 7).

By incorporating a virtual community into the course the intention was for the students to view the modules and skills taught in a patient-centred manner. Overall, we believe that NZ Pharmville facilitated patient-centred learning; this study found that the virtual community platform for teaching within the BPharm course was well received by students. The majority of students reported that the platform assisted with the development of empathy for the patients they encountered in the practical sessions, more so than the paper-based cases, which they received in concurrent courses. In addition, the students felt well prepared for their future role involving interacting with patients.

Previous research has also described a positive response from students to a virtual community and that students were able to “identify with various characters within the community” [18]. Marriott et al. (2012) also found a correlation between the degree of integration of Pharmville resources with the learning outcomes of an activity and the authenticity described by students [18]. Communities of virtual patients have also been used in nursing education [21–23]; initial reports have been positive from the students [24, 25]. However, none of these studies specifically explored students perceptions of whether their virtual community assisted in the development of empathy for patients. This study therefore highlights that a virtual community can help promote the development of empathy.

Other methodologies have been used to teach empathy to students. A study by Chen and colleagues (2008), described a method by which pharmacy students enrolled in an advanced course simulated the life of an actual patient for 10 days as part of an assignment which aimed to improve their empathy toward underserved populations [26]. The authors report that the assignment increased empathy for patients with different backgrounds and differing medical and psychosocial challenges [26]. Clinical education research has also shown the benefits of reflective practice, and the need to revisit skills, as per Kolb’s learning cycle [8]. Fuhrman and colleagues (2001) also emphasise the need to build realistic expectations of pharmacy practice for students in teaching and provide opportunities to increase confidence in dealing with all patient types [27]. NZ Pharmville allows for the students to repeatedly access the virtual community via ongoing activities in practical sessions. The benefits of repeated exposure were reported in the study by Marriott et al. (2012), whereby students confirmed the development of familiarity over years 1 to 4 [18]. Giddens (2010) found that the benefits of their virtual community increased over time with repeated use [25].

Virtual communities have been described as a way to allow academics to bridge the gap between theory and application [18]. By incorporating a virtual community into our teaching we hoped to provide the opportunity for ongoing practice and reflection of core skills [8]. In addition, students have opportunities to broaden their exposure to a range of patients over the duration of the course, which enabled them to practice adapting core skills to different situations. We believe that this teaching platform provides appropriate facilitation of a realistic teaching environment to encourage empathy and patient-centred care.

Students reported that NZ Pharmville helped them prepare for practical sessions. This was shown in both the Likert-response items and free-text response questions. Specifically many students felt that having the vignettes available prior to the sessions and also having all the cases within the practical sessions based on these families helped them with their preparation. Within the free-text responses, some students reported that having to watch the vignettes prior to the lab, added to their workload. In a study that used virtual learning technology, Smith et al. (2014) reported that while students had the opportunity to practice skills outside of the classroom, less than one third of the cohort did [28]. In this study a few students suggested that if they were able to watch these clips at the start of the practical sessions, this would allow them to prepare for their in-class activities, and also reduce their pre-practical session workloads. This is something that could be considered in the next iteration of our teaching.

Whilst in general student feedback demonstrated that NZ Pharmville was well received, there were a substantial proportion of students who gave a neutral Likert scale response and many supplied no responses to the free text questions. This survey was completed at the end of the course to allow for the maximum use of the virtual community prior to the evaluation. Unfortunately this also coincided with a number of assessments occurring during the final week which could have influenced student attitudes and responses. Future research could evaluate the intervention earlier or at different time points throughout the semester.

One other limitation of this study was that we could not directly compare the responses to these questions with students who had not had NZ Pharmville integrated into the course. However, students have been exposed to paper-based case studies in other parts of the BPharm; item 11 of this current research asks students to compare their development of empathy from the videos of patients in NZ Pharmville with reading a patient description. This question received a 65 % positive response from the students.

This was a cross-sectional study to gauge student use and perceptions of NZ Pharmville. Now we have established its acceptability to students we plan to evaluate the effectiveness of the platform for evoking deeper learning and evaluate its impact on core skills. We plan to do this with future student cohorts possibly using one of the assessment methods detailed by Bray and colleagues (2011) [29]. In addition we will expand the number of patients in the virtual community with the intention of integrating the resource throughout the whole pharmacy curriculum.

## Conclusion

This study aimed to evaluate student perceptions of a virtual community integrated into the third year of the Bachelor of Pharmacy degree at The University of Auckland. The use of virtual communities, such as that of NZ Pharmville, show promise as a platform to aid in teaching and learning. The resources in NZ Pharmville allow students ongoing access to patient video clips that attempt to mirror a real life situation, and enable students to engage with the fictional characters. Overall the students’ perceptions of NZ Pharmville were positive, with the majority of students finding the community a helpful way to prepare for practical sessions, for helping develop their interview and counselling skills within a role-play scenario and increase their empathy for patients, all of which are important skills for their future roles as pharmacists. This teaching method appeared to promote active patient-centered learning and allowed students to reflect on and revisit these skills on a weekly basis. Future work will expand the number of patients in the virtual community with the intention of integrating the resource throughout the curriculum.

More research is required to assess whether the empathy development and other patient-centered skills of the students are increased, improved and persist and how often the students watch the video clips.

## Abbreviations

ACPE: Accreditation Council for Pharmacy Education
CAPE: Centre for the advancement of pharmacy education
